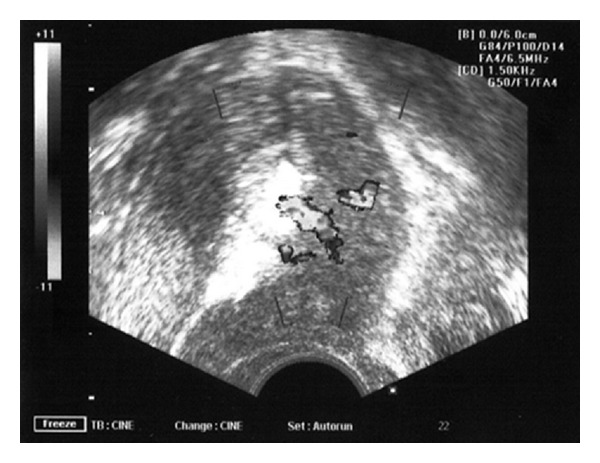# Postpartum Uterine Wound Dehiscence Leading to Secondary PPH: Unusual Sequelae

**DOI:** 10.1155/2012/154685

**Published:** 2012-06-07

**Authors:** Rinku Sengupta Dhar, Renu Misra

**Affiliations:** Department of Gynecology and Obstetrics, Sitaram Bhartia Institute of Science and Research, B-16, Qutab Institutional Area, New Delhi 110 016, India

## Abstract

Secondary postpartum haemorrhage due to partial or complete dehiscence of uterine wound after caesarean section is unusual. Authors present here a patient with secondary postpartum haemorrhage following uterine dehiscence after caesarean delivery. Conservative management failed to control the bleeding, and she eventually needed hysterectomy. All women who have significant PPH following caesarean should undergo evaluation for any defect in the scar. Scar dehiscence has been diagnosed and repaired after many years of caesarean section in women with persistent abnormal bleeding. Therefore, this condition may have long-term implication if missed postpartum.

## 1. Introduction

Secondary postpartum haemorrhage (PPH) after caesarean occurs in about 1 : 365 cases [1]. The most common etiological factors are retained products of conception and subinvolution of the placental site. A rare cause is partial or complete dehiscence of the lower uterine segment incision [1]. The patient may present with excessive vaginal bleeding and pelvic pain as early as 11 days to as late as 12 weeks after surgery [2]. She may also present with dysmenorrhoea and intermenstrual bleeding a few years after the caesarean section (CS) [2]. We present here a patient with secondary PPH following uterine dehiscence after caesarean delivery who was managed conservatively initially but eventually needed hysterectomy.

## 2. Case Report

A 27-year-old para 2 with two previous caesarean sections was referred to us with postpartum heavy bleeding with passage of clots for one day. There was a history of similar episode 2 weeks back when she was managed conservatively with 3 units of packed cells and intravenous antibiotics. There was no history of fever, unhealthy vaginal discharge, or wound infection. She had an elective caesarean section 10 weeks back. The caesarean section was done for thin scar (1 mm) detected on routine ultrasound.

**Figure 1:**
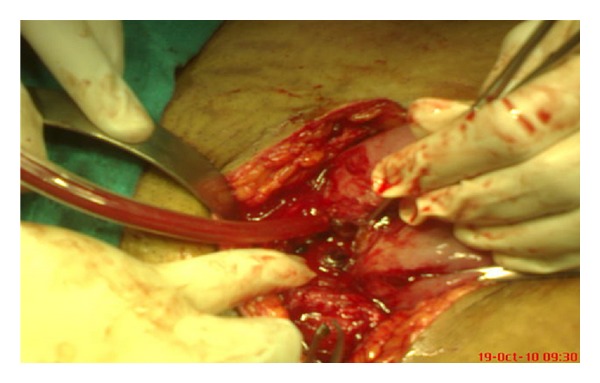


On examination, there was lower abdominal tenderness with no guarding or rigidity. Abdominal scar was healthy. No abdominal mass was palpable. On pelvic examination, uterus was bulky and OS was closed. There was active bleeding. Ultrasound showed an endometrial thickness of 13 mm and an anechoic lesion of 33 × 28 × 33 mm in lower uterine segment in the left lateral wall with high velocity blood flow on doppler in the surrounding myometrium (Figure 1). Her haemoglobin was 8 gm%. Platelet count and coagulation profile were within normal limits. A pelvic angiography to exclude uterine artery aneurysm was planned. But she had sudden acute hypotension with heavy vaginal bleeding and was thus taken up for emergency laparotomy. At laparotomy, there was minimal haemoperitoneum. The uterus was enlarged to about 10–12 weeks pregnant size. On dissecting the bladder down with blunt and sharp dissection, a complete dehiscence of the entire lower segment uterine incision was identified (Figure 2). An active arterial bleed was seen at the left angle of the incision. Bladder wall was intact. The margins of the incision were unhealthy and necrosed, and therefore a decision to proceed for a total abdominal hysterectomy was taken. A swab for culture and sensitivity was taken from the margins of the uterine incision which later revealed significant growth of *E. coli*. She received 6 units of packed cells and 4 units of fresh frozen plasma (FFP). Her postoperative period was uneventful and she was discharged on the fifth postoperative day.

## 3. Discussion

Secondary PPH following caesarean section is uncommon. The traditional causes of secondary PPH which typically are retained placental fragments are less likely to arise after caesarean section because delivery of the placenta is directly observed. The other causes are subinvolution of placental site, fibroids, infection, gestational trophoblastic disease, and rarely AV malformation. Severe PPH due to partial or complete dehiscence of uterine wound is unusual and the bleeding is probably due to eroded vessels on the uterine margin as was seen in our case.

Reported risk factors are nulliparity, diabetes, emergency surgery, infection, and incision placed too low in the uterine segment [1].

Supra pubic tenderness suggested possible endomyometritis in our case. This case report points out that it is also important to consider uterine dehiscence especially if clinical findings suggest localised pelvic tenderness or pelvic abscess. Whenever the clinical and ultrasound findings do not suggest retained placental tissue, further investigations should be carried out. It is important not to get tempted to do an uterine curettage which can further damage the nonhealing uterine wound. Whereas a routine transvaginal ultrasound may show only fluid collection or haematoma in the scar area, a 3D ultrasound may identify dehiscence better [3]. An MRI with a heavily T2 weighted image may show a bright fluid filled tract [3]. A power Doppler imaging will be additionally useful to distinguish uterine pseudoaneurysm and A-V fistulas [3]. It will usually show characteristic blood flow pattern in these situations [4]. The Doppler findings of our case indicated the possibility of a uterine artery pseudoaneurysm, but the blood flow was not seen very distinctly in the cystic lesion at the scar area. A beta HCG may be additionally helpful to rule out choriocarcinoma. When a complete dehiscence is suspected or the patient is unstable or in presence of fulminant infection, it is better to go for exploratory laparotomy directly. Otherwise, pelvic arteriography may be recommended to confirm the presence of acquired vascular malformations [4]. In the same sitting, embolisation may be therapeutic in absence of major infection [4]. On exploratory laparotomy, the uterine incision may appear healthy or necrotic. The dehiscence of a fresh caesarean section may be associated with an acute infection. Infectious necrosis and endomyometritis may be also present [5]. In our case, margins were unhealthy and culture report showed *E. coli*. Suture material reaction, haematoma, and retrovesical haematoma have all been implicated in the dehiscence of uterine incision, but in our case it probably was preexisting infection [5]. Conservative resuturing after debridement can be done but if the margins of the wound are infected or if there is a marked endomyometritis or intraabdominal abscess, hysterectomy is preferred [5]. There are reports of conservative surgery even with infection [2]. In this case, the culture detected corynebacterium and prevotella, but the wound was surgically repaired with broad spectrum antibiotic cover.The consequences of this complication for a future pregnancy is unknown [1]. It has been recommended that all women who retain their uterus after a significant PPH following CS should undergo evaluation for any defect of scar [1]. Laparoscopic and vaginal repair of scar dehiscence has been diagnosed and repaired after many years after caesarean section [6, 7]. Therefore, this condition if missed immediate postpartum can have long-term implication.

## Figures and Tables

**Figure 2:**